# Stably integrated and expressed retroviral sequences can influence nuclear location and chromatin condensation of the integration locus

**DOI:** 10.1007/s00412-012-0366-9

**Published:** 2012-03-14

**Authors:** Jens Nagel, Birgit Groß, Manja Meggendorfer, Carolin Preiss, Manuel Grez, Ruth Brack-Werner, Steffen Dietzel

**Affiliations:** 1 Department Biologie II, Ludwig-Maximilians-Universität München, Großhaderner Str. 2, 82152 Planegg-Martinsried, Germany; 2 Institute of Virology, Helmholtz-Zentrum München, German Research Center for Environmental Health, Ingolstädter Landstrasse 1, 85764 Neuherberg, Germany; 3 Georg-Speyer-Haus, Paul-Ehrlich-Straße 42-44, 60596 Frankfurt am Main, Germany; 4 Walter-Brendel-Zentrum für Experimentelle Medizin, Ludwig-Maximilians-Universität München, Marchioninistr. 27, 81377 Munich, Germany

## Abstract

The large-scale chromatin organization of retrovirus and retroviral gene vector integration loci has attracted little attention so far. We compared the nuclear organization of transcribed integration loci with the corresponding loci on the homologous chromosomes. Loci containing gamma-retroviral gene transfer vectors in mouse hematopoietic precursor cells showed small but significant repositioning of the integration loci towards the nuclear interior. HIV integration loci in human cells showed a significant repositioning towards the nuclear interior in two out of five cases. Notably, repositioned HIV integration loci also showed chromatin decondensation. Transcriptional activation of HIV by sodium butyrate treatment did not lead to a further enhancement of the differences between integration and homologous loci. The positioning relative to splicing speckles was indistinguishable for integration and homologous control loci. Our data show that stable retroviral integration can lead to alterations of the nuclear chromatin organization, and has the potential to modulate chromatin structure of the host cell. We thus present an example where a few kb of exogenous DNA are sufficient to significantly alter the large-scale chromatin organization of an endogenous locus.

## Introduction

The mammalian interphase nucleus is a highly organized and compartmentalized organelle in which each chromosome occupies its own territory, providing the functional form of chromatin (Cremer et al. 2006; Lanctôt et al. 2007; Meaburn and Misteli 2007; Cremer and Cremer 2010). Chromosome territories themselves also have a substructure with distinct subdomains for chromosomal subregions (Dietzel et al. 1998). The radial nuclear positioning of chromosome territories is non-random. In many cell types, gene-rich territories and chromosome regions preferentially occupy more internal regions while gene-poor territories and heterochromatin are preferentially at the nuclear periphery (Croft et al. 1999; Boyle et al. 2001; Cremer et al. 2001). Other studies showed that GC-rich chromosome regions are more likely to occur in central positions than GC-poor regions (Hepperger et al. 2008; Küpper et al. 2007).

Thanks to their capacity to deliver genetic material into target cells, viral gene vectors play an important role in the field of gene therapy. Retroviral vectors integrate stably into the host genome and therefore have the potential to exert enduring therapeutical effects (Kay et al. 2001; Mancheno-Corvo and Martin-Duque 2006; Edelstein et al. 2007). Until July 2011 1714 gene therapy clinical trials were approved worldwide (http://www.wiley.com/legacy/wileychi/genmed/clinical/) with retroviral vectors coming in a close second (23% including lentiviral vectors) after adenoviral vectors (24%). Retroviral gene transfer vectors lack most retroviral protein coding sequences while retaining the viral packaging signal and the 5′ and 3′ terminal repeat sequences (LTRs), which are required for DNA integration (Thiel and Rössler 2007; Nolan 2009). Integration potentially may lead to oncogenesis by disruption of tumor suppressor genes or activation of nearby proto-oncogenes and is thus a reason for concern (Hacein-Bey-Abina et al. 2008; Howe et al. 2008; Ott et al. 2006; Stein et al. 2010).

Retroviruses, in particular HIV, are also important human disease agents. For both, retroviruses and retroviral vectors, it is not clear how the genomic site for integration is determined, although some preferences were described (Bushman et al. 2005; Cattoglio et al. 2010; Cassani et al. 2009; Felice et al. 2009). HIV favors integration in transcribed chromosomal regions, thus improving chances for efficient expression of the viral genes (Wang et al. 2007). The only study on large-scale chromatin organization of retroviral integration loci we are aware of described an integrated, inactive HIV-1-derived gene vector associated with heterochromatin in about 10% of cells of a human lymphoid cell line and a loss of this association for the activated vector (Dieudonne et al. 2009). To our knowledge, an investigation of the impact of retroviral integration on nuclear positioning of the host loci by a comparison to the same loci without integration was not previously performed.

We studied the transcribed retroviral integration loci in three human cell types infected with HIV, astrocytes, HeLa cells and T-lymphocytes, as well as in a mouse hematopoietic precursor cell line transduced with a retroviral vector. Integration sites were mapped and their three-dimensional position was compared to the respective site on the homologous chromosome after fluorescence in situ hybridization (FISH) and confocal microscopy. Among other changes, we found that HIV integrations in HeLa cells were located significantly more interior in the nucleus than their homologous loci. For some transgenes, a repositioning towards more internal nuclear regions was described upon transcriptional activation (Dietzel et al. 2004; Tumbar and Belmont 2001), and similar findings were made for several gene loci and chromosomal subregions (Williams et al. 2006; Chuang et al. 2006; Zink et al. 2004).We therefore tested whether an artificial increase of HIV transcription by sodium butyrate induction (Quivy et al. 2002) would lead to even stronger differences between integration and homologous loci. Since HIV RNA is multiply spliced (Tazi et al. 2010), we also considered the possibility that the transcribed integration site attracts large numbers of splicing factors, resulting in a colocalization with signals obtained with anti-SC-35 splicing factors, so called speckles. We therefore investigated the positioning of HIV integration loci and homologous loci relative to SC35 splicing speckle surfaces.

## Materials and methods

### Cells

Generation of the mouse hematopoietic precursor cell line cloneB and definition of retroviral integration sites is described in (Modlich et al. 2006). Cells were cultivated in IMDM medium supplemented with mIL-3 (final concentration 10 ng/ml), mSCF (50 ng/ml), fetal bovine serum (10%), 100 U/ml Penicillin and 100 μg/ml Streptomycin.

TH4-7-5 cells were established by HIV-1 infection of the human glioma cell line 85HG-66 derived from astrocytoma brain tumors (Brack-Werner et al. 1992). LC5-HIV cells were established by HIV-1 infection of the cell line L-132 (Mellert et al. 1990), which originally was thought to be derived from embryonic lung tissue but subsequently was identified as a HeLa derived cell line, according to LGC Standards (http://www.lgcstandards-atcc.org/LGCAdvancedCatalogueSearch/ProductDescription/tabid/1068/Default.aspx?ATCCNum=CCL-5&Template=cellBiology). KE37/1-IIIB cells were established by HIV-1 infection of the T-lymphoma derived cell line KE37/1 (Popovic et al. 1984). In the latter two cases, the cultures contain a pool of cells originating from several founders with various HIV integrations. By FISH (see below), we could visualize between zero and four integration sites per nucleus. All three cell types were cultured in RPMI Medium (supplemented with 10% FCS, 100 U/ml penicillin and 100 μg/ml streptomycin). For simplicity, subsequently cells are identified by their cell type only: astrocytes (for TH4-7-5), HeLa cells (LC5-HIV) and T-lymphocytes (KE37/1-IIIB), respectively.

Sodium butyrate (NaB) treatment was performed for 24 h at 0.5 mM. For controls and treated cells, the amount of HIV RNA was determined and compared to the amount of RNA polymerase II RNA as a control. To this end, quantitative polymerase chain reaction (qPCR) was performed on a LightCycler 480 (Roche) with the Light Cycler 480 SYBR Green I Master PCR kit.

### Integration site mapping

Retroviral integration sites in cloneB were analyzed as described (Modlich et al. 2006). To obtain HIV integration sites ligation-mediated polymerase chain reaction (LM-PCR) was accomplished using the Genome Walker Universal Kit (BD Biosciences Clontech, Palo Alto, CA, USA). HIV-1 LTR sequence specific outer primer (5′-AAAGGTCAGTGGATATCTGATCCCTGGCCC-3′) and inner primer (5′-CAGGGAAGTAGCCTTGTGTGTGGTAGATCC-3′) for nested PCR were applied using the PCR Kit advantage 2 (BD Biosciences Clontech). PCR products were purified (Quiaquick Gel extraction Kit; Quiagen, Hilden, Germany) and sequenced (Sequiserve, Vaterstetten, Germany; GATC Biotech AG, Konstanz, Germany). Integration sites were mapped by blasting the sequencing results on the NCBI homepage (http://blast.ncbi.nlm.nih.gov/Blast.cgi; version Build 36.2). In case of positive blast hits the “cytoview” display of the Ensembl Genome Browser (http://www.ensembl.org/index.html; version Ensembl 43) was used to select suitable bacterial artificial chromosome (BAC) clones that cover the genomic region around the integration locus.

### Preparation of cells for 3D-FISH

Astrocytes and HeLa cells were seeded on coverslips at 70–80% confluence. Since T-lymphocytes and cloneB cells grow in suspension, growing cells were attached to poly-l-lysine coated coverslips. All cells were fixed and prepared for 3D-FISH as described (Solovei et al. 2002; Hepperger et al. 2007). Briefly, T-lymphocytes and cloneB cells were incubated in 0.3× PBS for 45 s and subsequently fixed in 4% paraformaldehyde in 0.3× PBS for 10 min. Astrocytes and HeLa cells were fixed in 4% paraformaldehyde in 1× PBS for 10 min. All cells were permeabilized 15 min in 0.5% Triton-X100, incubated over night in 20% glycerol, subjected to five freeze–thaw cycles with liquid nitrogen, incubated in 0.1 M HCl for 6 min and stored in 50% formamide/2× SSC at 4°C until use (at least 48 h). In previous work, we could show that this procedure provides good structural preservation of the large-scale chromatin structure (Kim et al. 2007; Hepperger et al. 2007).

### Probes and fluorescence in situ hybridization

FISH probes for integration loci were generated from BAC clones ordered from BAC-PAC Resource Centre (Oakland, CA, USA; http://bacpac.chori.org). BAC DNA was extracted with the High Pure Plasmid Isolation Kit (Roche, cat. No. 11754777001). As HIV FISH probe, the pNL4-3 plasmid (Adachi et al. 1986) was used, isolated from E.coli (Plasmid isolation kit, Macherey-Nagel, Düren, Germany). Prior to labeling all FISH probes were amplified with the GenomiPhi™ V2 DNA Amplification Kit (GE Healthcare, Munich, Germany). BAC DNA was labeled with dinitrophenol (DNP)-deoxyuridine triphosphate (dUTP), digoxigenin-dUTP, biotin-dUTP or Texas Red-dUTP by nick translation as described elsewhere (Cremer et al. 2008). BACs were tested for correct chromosomal location by FISH on metaphase spreads together with the respective chromosomal libraries. HIV probe DNA was labeled with digoxigenin-dUTP or Cy3-dUTP by nick translation.

Hybridization was performed as described in (Hepperger et al. 2007). Haptens were detected with antibodies in blocking solution at 37°C, 45–60 min for each layer: rabbit-anti-DNP (1:200; Sigma-Aldrich, Deisenhofen, Germany), goat-anti-rabbit-Alexa488 (1:200, Molecular Probes (Invitrogen), Karlsruhe, Germany), goat-anti-rabbit-Cy3 (1:200; Dianova, Hamburg, Germany), AvidinAlexa488 (1:200; Molecular Probes), mouse-anti-dig-Cy5 (1:100, Dianova), goat-anti-mouse-Cy5 (1:100, Dianova). SC35 splicing speckles were detected with a mouse antibody (1:100, Sigma-Aldrich). 4′,6′-Diamidino-2-phenylindole (DAPI; Sigma-Aldrich) was used as DNA counterstain, VectaShield (Vector, Burlingame, CA, USA) was used for mounting.

### Confocal microscopy, image processing

**Fig. 1:**
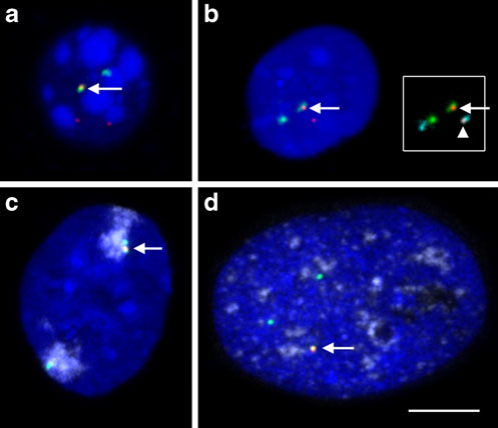
3D FISH on structurally preserved cell nuclei. **a** Mouse hematopoietic precursor cell cloneB. Three RNA transcribing integration sites (*red*) were detected, one of them (*arrow*) colocalizing with the BAC signal (MMU12D2, *green*). The other two RNA labeled integration sites were not evaluated in this nucleus. **b** Human T-lymphocyte. One FISH signal from the 2q11.2 BAC (*green*) colocalized with a HIV RNA signal (*red*, *arrow*). The 2q14.2 BAC (*cyan*) is shown in the inset only, together with the other FISH signals. One of them colocalized with the second HIV signal (*arrowhead*). From FISH on metaphase spreads both integration sites are known to be on the same chromosome 2. **c** HeLa cell, BAC and HIV signals as before, X-chromosome paint probe in light blue. **d** Human astrocyte, BAC and HIV signals as before. SC35 splicing speckles in light blue. **a**–**c** Projections of confocal image stacks, **d** projection of three adjacent sections. Dark blue — Dapi-stained DNA; scale bar 5: μm for all four images

Three-dimensional confocal image stacks were recorded on a Leica TCS SP5 microscope with a 63× oil immersion objective. Voxel size was 80 nm in *xy* and 240 nm in *z*. The software ImageJ (http://rsb.info.nih.gov/ij/) was used for shift correction of chromatic aberration and for setting signal thresholds for subsequent computerized image analysis. The intensity of HIV and gene vector signals was weak compared to BAC signals. Hence, for simultaneous presentation of color channels in Fig. 1, the HIV or gene vector channel was strongly enhanced with the Brightness/Contrast function in ImageJ.

The surface area of BAC signals was measured in ImageJ after noise reduction (Gaussian filter, Sigma (Radius) = 1) with the object counter 3D plug-in (Bolte and Cordelieres 2006). Subjective influence was minimized by normalizing the BAC signals with the stack normalizer plug-in and by applying a constant threshold of 50 (HeLa cells) or 100 (T-lymphocytes).

For SC35 experiments, light optical sections were deconvolved by the Huygens software package (Scientific Volume Imaging B.V., Hilversum, Netherlands) using measured point spread functions.

**Fig. 2:**
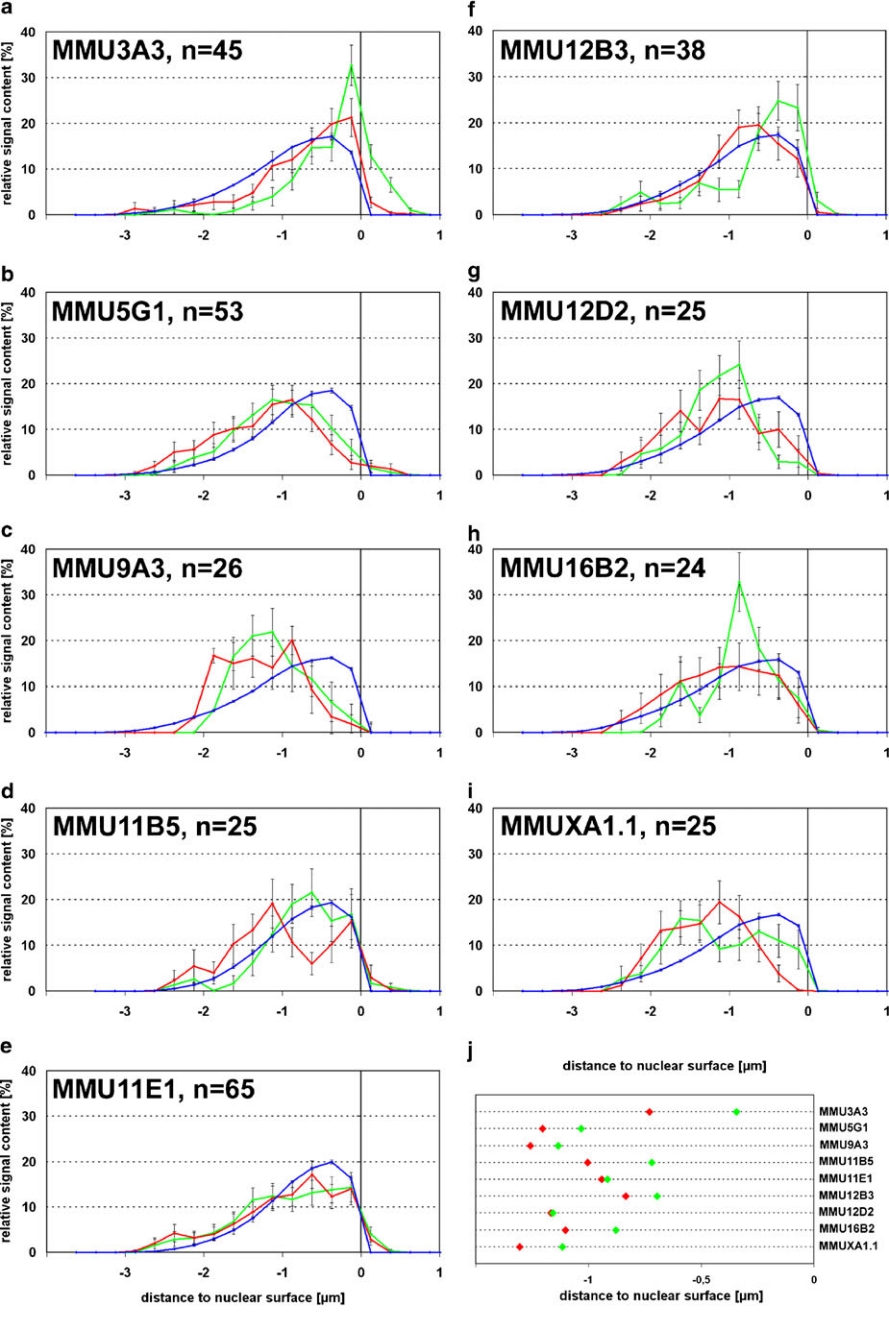
Nuclear distribution of BAC signals in mouse hematopoietic precursor cells. **a**–**i** *Green* BAC signals not colocalizing with gene vector signals; *red* BAC signals colocalizing with gene vector signal; *blue* Dapi stained DNA. Distances to the nuclear surface are given in μm, negative values reflect signals inside nucleus and positive values those outside the nucleus. **j** Mean values of medians of respective BAC distribution curves for each integration locus; *green* BAC signals not colocalizing with gene vector signals; *red* BAC signals colocalizing with gene vector signal

3D distance measurements from BAC signal voxels to the nearest surface of the nucleus or SC35 splicing speckles were performed with the ADS program (absolute distance to surface) as described by Küpper et al. (2007). Briefly, thresholds were interactively set to allow the program to calculate for each voxel of a signal the shortest 3D distance to the nuclear surface. To minimize bias, all thresholds for a data set were determined by one person in one go. Reevaluation of such data sets by other persons generally resulted in a very similar outcome. Integration loci and control loci were always evaluated in the same cells. Intensity-weighted frequencies were collected in classes with 250 nm width as percentage of the total given signal in a nucleus and the averages over the population of nuclei were calculated. Due to the limited microscopic resolution the transition from DNA signal to background is not sharp but blurred and the position of the nuclear surface varies somewhat with the applied threshold. Thus, depending on the threshold, peripheral FISH signals may come to lie partially outside the nucleus. For example, in Fig. 2a, the external portion of the control locus is composed entirely of such peripheral signals which are partially inside and partially outside. Since the same defined surface was used for all signals in the nucleus, the comparison of integration loci with control loci is not affected by the blurred surface. Graphs were generated in Microsoft Excel. Publication figures were assembled in Adobe Photoshop.

### Statistical evaluation

We generally used the paired *t*-test, a pair consisting of the values for the integration site and the homologous site (averaged when two were present: HeLa18q22.3, Astro18q22.1) from the same nucleus. For radial nuclear positioning, the median values were used. Some distributions were not normally distributed, we thus had to apply the Wilcoxon signed rank (WSR) test for paired samples instead (radial distributions of MMUXA1.1, HeLa18q22.3, HeLaXq22.1, Astro18q22.1, surface pixels HeLa18q22.3). Distributions in untreated and sodium butyrate treated cells were compared with the Mann–Whitney rank sum test. All calculations were performed with SigmaStat 3.5 (SPSS, Chicago, IL, USA).

### Databases and genomic properties

The gene density in a 0.5-, 2- and 10-Mbp window around the integration site was read off the NCBI Map viewer (http://www.ncbi.nlm.nih.gov/mapview/, version Build 37.1). The sequence was downloaded from there and submitted to RepeatMasker (version open-3.2.9, http://www.repeatmasker.org/) to obtain the percentage consisting of GCs and of repetitive elements

## Results

### Experimental strategy

To study the 3D localization of retroviral integration loci, we first mapped integration sites by LM-PCR and used this sequence information to identify the integration loci (Tables 1 and 2). To visualize integration loci and their homologous regions microscopically, we used BACs covering the mapped chromosomal loci as FISH probes. BACs were first tested for correct genomic localization by FISH on metaphase chromosome spreads of the respective cell line. The karyotype was analyzed on the same preparations for chromosomal translocations or other rearrangements. If an integration harboring chromosome differed from its homolog, the respective integration locus was excluded from further analysis. For 3D FISH experiments on structurally preserved nuclei, we used HIV proviral DNA (HIV infected cells) or retroviral vector DNA (mouse cells) in addition to BACs as a FISH probe (Fig. 1). Thus, integration loci were colabeled by a BAC signal and a vector signal while the homologous regions were labeled by BAC signals alone. Despite the low intensity of the HIV FISH signal (see methods), it was still much stronger in 3D FISH preparations than expected for a ~10-kb sequence (Fig. 1). Control experiments with RNase revealed that this was due to hybridization of the labeled probe to HIV RNA. Thus, only transcribed HIV integration sites were evaluated in our study. After confocal microscopy, quantitative analysis of the 3D radial nuclear position of integration and control loci was performed with the BAC signals only. To avoid a potential bias due to differences in FISH signal appearance of BAC and HIV or vector signals, HIV and vector signals were used for identification of the integration locus but not for 3D evaluation.

**Table 1:** Overview of gene vector integration loci in the mouse stem cell line “cloneB”

Integration locus	MMU 3A3^+^	MMU 5 G1^+^	MMU 9A3	MMU 11B5	MMU 11E1^+^	MMU 12B3^+^	MMU 12D2	MMU 16B2	MMU XA1.1^+^
Nearest gene	Evi1	Mad1like1	Dnm2	Lig3	Gm885	Akap6	Batf	AI480653	Wdr45
Distance to nearest gene	121 kb	0	0	2.6 kb	0	0.4 kb	13.6 kb	0	0.4 kb
Gene ID	14013	17120	13430	16882	380732	238161	53314	268880	54636
BAC name	RP23-439N22	RP23-217N11	RP23-317E10	RP23-316C11	RP23-247J12	RP24-267H3	RP23-369N11	RP24-206H1	RP23-54C14
Gene density									
0.5 Mb	4	18	40	24	20	4	32	6	52
2 Mb	9	21	39.5	24	22	6.5	21.5	12	33
10 Mb	7.2	23.4	17.3	18.7	17.4	8.5	17.1	10.3	18.8
chromosome	9.9	11.6	13.2	17.2	17.2	11.3	11.3	9.9	11.1
GC content									
0.5 Mb	39.9%	51.3%	49.1%	46.9%	45.3%	41.1%	45.1%	46.7%	38.4%
2 Mb	40.6%	50.5%	47.9%	45.3%	45.1%	41.1%	45.9%	45.4%	43.1%
10 Mb	41.5%	47.9%	41.3%	44.8%	44.5%	40.2%	43.9%	43.2%	41.9%
chromosome	40.5%	42.5%	43.0%	44.0%	44.0%	41.5%	41.5%	41.0%	39.0%

Integration locus description defines the chromosomal band; Loci marked by + have been described before (Modlich et al. 2006); gene ID from NCBI mouse genome database; BAC name identifies the BAC used as a probe in FISH experiments. Gene density is given as genes per megabase, calculated for 0.5, 2 and 10 Mbp windows around the integration site, and for the whole chromosome. The GC content in percent is given for the same windows

**Table 2:** HIV integration loci in three investigated cell lines, HeLa cells, T lymphocytes (TLy) and astrocytes (Astro)

Integration locus	HeLa 6p12.3	HeLa 11q22.3	HeLa 15q21.3	HeLa 16p13.3	HeLa 18q22.3	HeLa Xq22.1	TLy 2q11.2	TLy 2q14.2	Astro 18q22.1
Nearest gene	SUPT3H	PDGFD	ZNF 280D	AXIN1	NETO1	CSTF2	KIAA 1310	PTPN4	CDH19
Distance to nearest gene	0	250 kb	0	0	700 kb	0	0	0	0
Gene ID	8464	80310	54816	8312	81832	1478	55683	5775	28513
BAC name	RP11-818O21	RP11-63H12	RP11-566D24	RP11-517F15	RP11-693I21	RP11-255J06	RP11-67L23	RP11-132N24	RP11-831H17
Chromosome integrity	−	−	−	−	+	+	+	+	+
sequence	GCAAGTTTTCAGTTACTGTGCTTGATAGGC	AAATCATTAAGTTGTTAATGGAATTTTAAG	CAGAAAAATGCAAATAGGGTCATTTTAGTC	GGGCTTTCCCTAGGTTCTGTTCATCAGGTG	CATAGAGCAAGGGGCCAACAGACCCCCTTA	ACAGCACAACTGGATACCACTGAATTAATC	GGTGGGTCTGGACCACTCCTGGATTCTGGG	TGCCTTCCTCAGACTTTGTTACAGCATAGG	ATAACAATATTGTATAATATATGAAGAAAT
Gene density									
0.5 Mb	6	2	10	54	0	34	24	12	2
2 Mb	8.5	7.5	8.5	41.5	4	21.5	20	11	2
10 Mb	14.7	7.8	8.5	29.3	3.8	11.6	11.8	4.7	4.5
chromosome	11.5	15.3	12.0	14.8	6.7	10.5	9.1	9.1	6.7
GC content									
0.5 Mb	35.9%	35.8%	37.5%	55.3%	35.9%	41.7%	47.7%	39.5%	34.4%
2 Mb	41.7%	44.8%	39.3%	58.6%	38.4%	40.2%	44.9%	45.6%	34.8%
10 Mb	41.7%	37.2%	39.6%	53.6%	39.2%	38.5%	43.3%	40.0%	37.0%
chromosome	40%	42%	42%	44%	40%	39%	40%	40%	40%

Loci are identified by the chromosomal band in which they sit. A distance of 0 to the nearest gene means the integration is inside the gene. Gene ID is from the NCBI human genome database. BAC name identifies the BAC used as a probe in FISH experiments. Chromosome integrity was essential to compare the nuclear distribution to the homologous locus (see main text). “Sequence” identifies the start of the sequenced host genome from LM-PCR directly adjacent to the 5′ LTR of the integrated provirus. Gene density is given as genes per megabase, calculated for 0.5, 2 and 10 Mbp windows around the integration site and for the whole chromosome. The GC content in percent is given for the same windows

### Integration loci in a mouse hematopoetic precursor cell line were repositioned to more internal nuclear regions

The mouse hematopoietic precursor cell line “cloneB” was created by transduction with the LTR-driven retroviral gene vector pSF91-GFP (Modlich et al. 2006). In addition to five integration sites mapped in the original study, we identified four additional ones (Table 1). FISH on metaphase chromosomes with BACs and corresponding chromosomal paint probes did not reveal any aberrations from a normal karyotype. In any one nucleus, up to four integration sites gave rise to a detectable gene vector FISH signal, arguing that only a subset of sites was transcribed in individual nuclei. Analysis of the 3D radial distribution of the nine integration loci in structurally preserved nuclei (Fig. 1a) revealed that MMU3A3 showed a highly significant shift of the integration locus towards more internal nuclear regions when compared with the corresponding locus on the homologous chromosome (*p* < 0.001; Fig. 2a,j). Notably, MMU3A3 is in the immediate vicinity of the protooncogene Evi1 (ecotropic virus integration site 1), a region with frequently observed viral and gene vector integrations (Modlich et al. 2006; Ott et al. 2006; Wieser 2007; Metais and Dunbar 2008).

MMU5G1 showed a less pronounced but still significant difference (*p* = 0.033; Fig. 2b,j). For some of the other seven integrations loci the difference to the corresponding locus on the homologous chromosome was very small and none of them was significant (*p* > 0.05, *n* between 24 and 65; Fig. 2c–i). However, pooled values from these seven loci (*n* = 228) revealed a highly significant difference (*p* = 0.002; WSR test). In agreement with this result, the average median position for all nine integration loci was more internal than the respective homologous locus (Fig. 2j). Taken together, retroviral integration loci in this mouse hematopoietic precursor cell line show a more internal position than their respective homologous loci.

### HIV integration sites

Retroviral integration in human cells was investigated in a HeLa-, a T-lymphocyte- and an astrocyte cell line previously generated by infection with HIV (see methods) but with hitherto unknown genomic positions of integration sites. LM-PCR identified a total of nine integration sites, of which seven were within genes (Table 2). No evident preferences were found concerning GC content or gene density around the integration sites (Table 2).

FISH on metaphase chromosome spreads of the three cell lines revealed that five of the nine mapped integration sites were evaluable while four were on chromosomes involved in structural aberrations precluding a meaningful analysis. For example the genome of the HeLa cell line had three differently rearranged chromosomes 11, rendering a comparison of the integration site 11q22.3 with its homologous site futile. Metaphase spreads of HeLa cells also revealed that the two evaluable integration sites on chromosomes 18 and X did not occur together, suggesting that the cell population consisted of several subclones with independent integration events. The number of HIV-FISH signals in structurally preserved nuclei in HeLa cells was two (3%), one (74%) or 0 (23%); in T-lymphocytes four (7%), three (19%), two (34%), one (33%) or zero (7%); and in astrocytes one (95%) or zero (5%; *n* between 70 and 80), while undetected integration sites did not produce sufficient levels of RNA for the generation of a signal. For T-lymphocytes, this observation suggests that there are at least two more, yet unmapped, integration sites.

### Two of five HIV integration loci were repositioned and decondensed

**Fig. 3:**
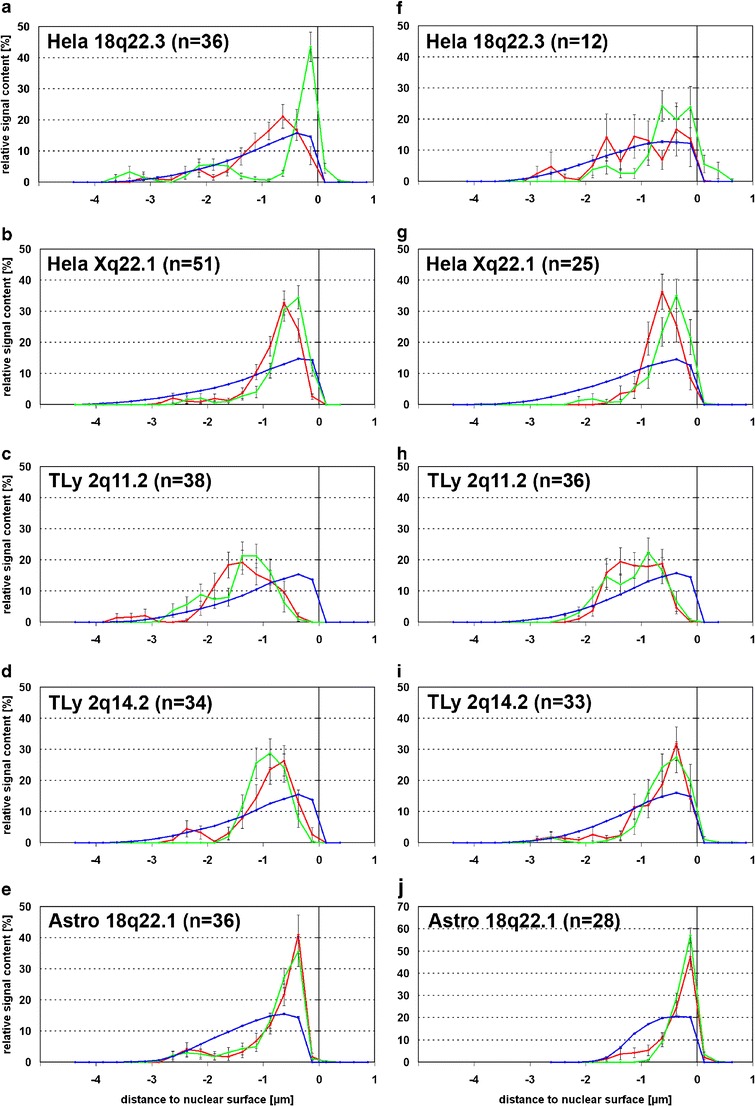
Nuclear distribution of BAC signals in HIV infected human cell types. **a**–**e** Untreated cells, **f**–**j** sodium butyrate-treated cells. *HeLa* HeLa cells, *TLy* T-lymphocytes, *Astro* astrocytes; *blue* DNA distribution, *green* distribution of BAC signals not colocalizing with HIV signal, *red* distribution of BAC signals colocalizing with HIV signal. Distances to the nuclear surface are given in microns (μm); negative values reflect signals inside nucleus and positive values those outside the nucleus

**Fig. 4:**
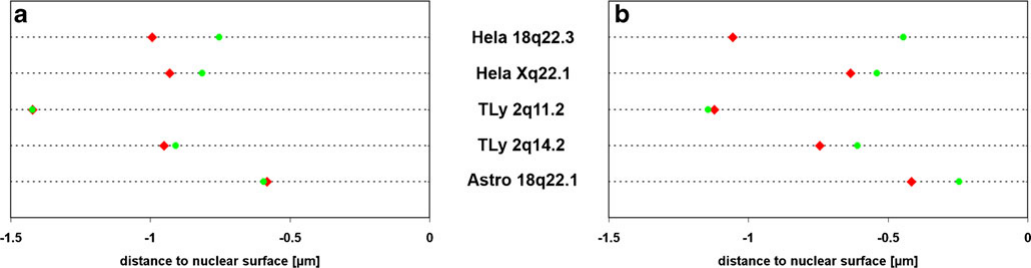
Mean values of medians of integration and control loci in HIV infected human cell types. In individual nuclei, the median position of a given BAC signal was determined. The mean values of those medians for HIV-colocalizing or not colocalizing signals over all nuclei are shown here. **a** Untreated cells. **b** Sodium butyrate-treated cells. *Green* mean value of medians of BAC distribution curves not colocalizing with HIV signal in respective cell type; *red* mean value of medians of BAC distribution curves colocalizing with HIV signal in respective cell type. For TLy2q11.2, the two values in **a** are so similar that the data points lay on top of each other

3D image analysis of radial nuclear positions revealed that the integration loci HeLa 18q22.3 and HeLa Xq22.1 (see Table 2) were highly significantly more internal than their homologous regions (*p* < 0.001 and *p* = 0.002, respectively; Figs. 3a,b and 4a). The integration loci TLy 2q11.2, TLy 2q14.2 and Astro 18q22.1 (Fig. 3c,d,e) did not show significant differences.

For the two significantly repositioned HIV integration loci, we also investigated the positioning relative to the surface of the harboring chromosome territories. Compared to their homologous loci, they did not show a significant difference in this analysis (*p* > 0.05). The nuclear radial position of the harboring chromosome territories was also unaffected.

**Fig. 5:**
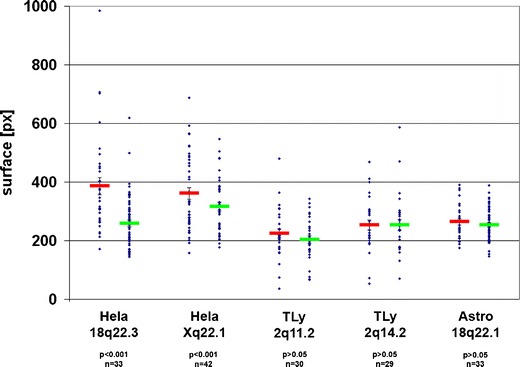
Surface area of BAC signals for HIV integration and homologous control loci. *Blue dots* surface area in pixels of all BAC signals; *bars* mean values of BAC surface area colocalizing (*red*) or not colocalizing (*green*) with HIV signal, respectively

Upon visual inspection of BAC signals, we noticed that in some cases signals of integration loci appeared larger and thus more decondensed than the BAC signals from the homologous chromosomes. 3D measurements of the signal surfaces confirmed this impression (Fig. 5). For HeLa 18q22.3, the BAC signal of the integration locus showed on average a 1.49 times larger surface (*p* < 0.001). For HeLa Xq22.1, the surface difference was smaller (1.14×), corresponding to a less pronounced repositioning (Figs. 3a,b and 4a), but still highly significant (*p* < 0.001). Both BACs did not generate a detectable signal when used as a probe for RNA FISH, arguing that the increased surface of DNA FISH signals is indeed due to chromatin decondensation and not to transcribed RNA from host sequences. For the other three integration loci, BAC signal surfaces did not show noticeable differences between integration and homologous loci (*p* > 0.05).

### Treatment with sodium butyrate increased HIV transcription but did not affect nuclear position of integration loci

Sodium butyrate is a deacetylase inhibitor which was shown to increase HIV-1 transcription (Quivy et al. 2002). This allowed us to investigate whether increased HIV transcription would enhance or cause positional differences between integrations and homologous loci. Sodium butyrate treatment indeed led to elevated HIV RNA levels, between twofold in T-lymphocytes and tenfold in HeLa cells. However, the elevated transcription of the integrated virus did not lead to a significant increase in positional differences between integration loci and homologous controls (Figs. 3f–j and 4b).

### HIV integration loci did not show altered position relative to SC35 splicing speckles

Production of the full array of HIV-1 proteins involves the production of multiple HIV-1 mRNA species by alternative splicing of a primary transcript (Tazi et al. 2010). SR proteins such as SC35 are serine/arginine-rich and known to be essential for alternative splicing (Lin and Fu 2007; Graveley 2000). For HIV RNA and SC35, both, colocalization (Favaro et al. 1998) and random distribution (Berthold and Maldarelli 1996; Boe et al. 1998; Zhang et al. 1996; Bell et al. 2001), was described for transfected cells. To our knowledge, only one study investigated this relation in infected cells, finding a random relative distribution (Bell et al. 2001). We tested for repositioning effects due to HIV integration relative to SC35 speckles, manifested by positioning differences between the integration locus and the homologous chromosomal region (Fig. 1d).

**Fig. 6:**
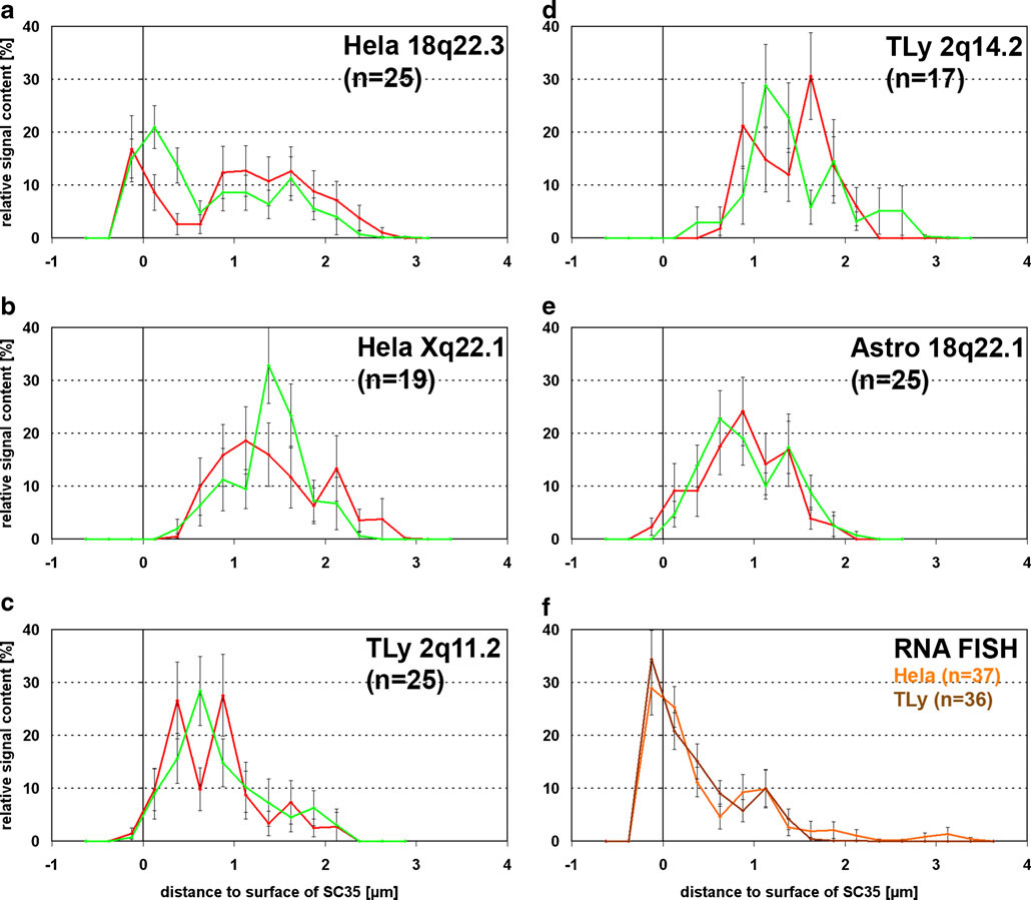
Distribution of BAC signals relative to SC35 splicing speckles. DNA FISH on HeLa cells (**a**, **b**), T-lymphocytes (**c**, **d**) and astrocytes (**e**). Distance to the closest surface of SC35 speckles in microns; negative values reflect signals inside speckles and positive values those outside speckles. *Green* distribution of BAC signals not colocalizing with HIV signals; *red* distribution of BAC signals colocalizing with HIV signals. **f** RNA FISH; *orange* distribution of HIV RNA signals relative to the surface of SC35 speckles in HeLa cells; *brown* same for T-lymphocytes

Generally, BAC signals of both, the integration loci and the homologous regions did not contact SC35 speckles, again with the exception of HeLa 18q22.3 where 15–20% of BAC signals colocalized with or were found adjacent to SC35 (Fig. 6a–e). Significant differences between the position of the integration locus and the homologous locus relative to SC35 speckles were not found (*p* > 0.05). By contrast, about 30% of the larger HIV RNA signals in HeLa cells and T-lymphocytes contacted or colocalized with SC35 speckles (Fig. 6f).

## Discussion

The current study provides new insights on the influence of stable retroviral integration on nuclear chromatin organization. In a hematopoietic mouse cell line, we found mostly modest but significant radial nuclear repositioning of transcribed retroviral vector integration sites to more internal regions. In two out of five transcribed HIV integration sites in human cell lines we also found significant repositioning and in addition indication of chromatin unfolding. An integrated retroviral DNA sequence of only a few kb in length thus altered large-scale chromatin structure of the host locus.

The extent of chromatin decondensation found in the two cases of HIV integration in HeLa cells was surprising. BACs used as FISH probes contained 150–200 kb genomic DNA. With about 10 kb, the size of the provirus is only 5–7%. As reflected by three of the HIV integration loci, such a small increase in base pairs by itself does not lead to a microscopically detectable size increase of the FISH signal from the surrounding host chromatin. Since the BAC only detects host sequences but not the provirus itself, larger FISH signals at HIV integration loci clearly indicate a decondensation effect on the neighboring chromatin. The larger FISH signals are not due to detection of RNA from host genes by the BACs. First, BACs did not produce a signal in RNA FISH and second, the BAC used for HeLa18q22.3 does not cover a gene since the distance from the integration site to the next gene is 700 kb.

Several studies comparing transcriptionally inactive and active chromatin found a more interior nuclear position for active chromatin (Zhao et al. 2009; Takizawa et al. 2008b; Kumaran et al. 2008; Lanctôt et al. 2007). A more internal radial position was also described for the active allele of a gene with monoallelic expression when compared to the inactive allele on the homologous chromosome in the same nucleus (Takizawa et al. 2008a). For an array of transgenes, we observed a more internal position as well as a decondensation after transcriptional activation (Dietzel et al. 2004). Taken together, such earlier data suggest that the changes in large-scale chromatin organization in the current study are also correlated to chromatin activation. In the current study, the largest change in radial position as well as decondensation was observed for the two investigated integration loci in HeLa cells, suggesting that also in this system decondensation and repositioning are connected. Chromatin decondensation and nuclear repositioning of transgene arrays was also found after chromatin activation but in the absence of transcription, showing that transcription itself was not required for chromosome reorganization in this particular case (Tumbar et al. 1999). It remains to be seen whether for the affected retroviral integration loci the nuclear repositioning is a prerequisite for or a consequence of transcriptional activation. Histone modifications may be involved in such a reorganizing, since viruses in general may cause chromatin modifications such as histone methylations, phosphorylations or acetylations (Lilley et al. 2007) but further studies are needed to clarify the mechanism behind our current and mentioned earlier observations.

The magnitude of induced large-scale chromatin reorganization by a given retroviral vector or virus may depend on the site of integration. Genomic properties such as gene density or GC content are obvious candidates but concerning these features neither the two repositioned HeLa loci nor the two strongest repositioned mouse loci stand apart from other investigated loci (Tables 1 and 2). It is conceivable that chromatin reorganization will lead to microscopically detectable changes only if the original chromatin environment of the integration locus is not sufficiently shaped for transcription, for example near peripheral heterochromatin. A reorganization of the locus might then occur to support transcription or as a consequence of it. While HIV was shown to preferentially integrate in actively transcribed regions (Wang et al. 2007), a large-scale chromatin reorganization might provide an additional mechanism to increase transcription probability. In accordance with this hypothesis a silent provirus of an HIV-1 derived gene vector in a lymphoid cell line was associated with pericentromeric chromatin regions in about 10% of investigated nuclei. After transcriptional activation this association was lost (Dieudonne et al. 2009). The HIV integration locus HeLa18q22.3 nicely fits in such a model since it repositioned away from the nuclear border, decondensed and lies in a gene free part of a gene poor chromosome (Table 2). The integration site at MMU3A3 is also repositioned away from the nuclear periphery and further away from the nearest gene than any other investigated mouse integration site. We suspected that sodium butyrate treatment, causing transcriptional upregulation of the integrated viral sequences, might cause an increase of differences between integration and homologous loci, but this was not the case.

In contrast to the results discussed in the previous paragraph, the second locus showing a significant repositioning in mouse cells, MMU5G1, starts from a comparatively internal position. Also, no repositioning was observed for Astro18q22.1 which is located at the periphery of the nucleus. This argues against a simple concept with reorganization when the integration locus is located at the peripheral heterochromatin. Additional rules need to be assumed to explain the observed phenomena. The fact that both HIV integrations showing large changes are from HeLa cells raises an alternative possibility, a cell type specific behavior. Some cell types, e.g., not terminally differentiated precursors or cancer cells, may be more permissive than others for chromatin rearrangements.

For the retroviral vector integration at MMU3A3 an interaction with the nearest gene, Evi1, is known, despite 120 kb in-between. The EVI1 (Ecotropic viral integration site 1) locus is one of the most frequent targets of retroviral integration, and in most known cases retroviral integration leads to overexpression of EVI1 (Buonamici et al. 2004; Metais and Dunbar 2008; Modlich et al. 2008, 2009). EVI1 is expressed in murine hematopoietic stem cells and downregulated during further differentiation (Kataoka et al. 2011). In hematopoietic precursor (lineage negative) cells, EVI1 overexpression was shown to enhance the self-renewal capacity and clonal dominance resulting from Evi1 upregulation was reported in several animal studies and human clinical trials (Li et al. 2002; Du et al. 2005; Ott et al. 2006; Laricchia-Robbio and Nucifora 2008; Bosticardo et al. 2009; Komeno et al. 2009; Stein et al. 2010). Noteworthy, retroviral integration at the EVI1 locus in cloneB resulted in a significant upregulation of EVI1 gene expression which was essential for immortalization and cell survival (Modlich et al. 2006). Thus the nuclear repositioning of the MMU3A3 integration locus is tied to activation of EVI1 and clonal immortalization.

Dieudonne et al. (2009) did not find a significant change in radial nuclear position upon transcriptional activation. In our study, investigated HIV proviruses were transcriptionally active, since HIV FISH signals contained large portions of detected HIV RNA. We compared the chromosomal locus containing the active provirus with the homologous chromosomal region without provirus integration. Mentioned study (Dieudonne et al. 2009) found the average distance to the nuclear border for nine integration loci in seven cell lines mostly between 1 and 2 μm, described as “closely at the periphery of the nucleus.” Our data also show that retroviral integration loci are mostly within 2 μm of the nuclear border (Figs. 2 and 3). However, comparison with the radial distribution of total nuclear DNA reveals that this range also contains the bulk of the chromatin, thus such a position is not necessarily skewed towards the nuclear border in a non-random way.

The finding that the two investigated integration loci in HeLa cells significantly changed their radial nuclear position but not their distance to the surface of their harboring chromosome territory while the radial nuclear chromosome territory position also did not change significantly may be surprising at first. Two possible explanations come to mind. First, the repositioned integration site may have taken a part of the chromosome territory with it that was large enough to keep the distance from the integration locus to the territory surface constant but which was too small to significantly affect the radial nuclear position of the whole territory. Second, the territory may have been remodeled in a way that the integrations locus kept the average distance to the territory surface but now was located in a part of the territory which was closer to the nuclear center.

While one might expect that integration and strong transcription of HIV within a gene poor region such as 18q22.3 might induce or attract a microscopically detectable accumulation of splicing factors to the site, we did not observe a change in the association frequency with splicing factor accumulations. Neither did any of the other HIV integration loci show a significant change in the average distance to splicing speckles. Consistent with this, HIV FISH signals were frequently found away from splicing speckles (Fig. 6f).

In conclusion, results from two independent experimental systems, HIV in human cells and gene vector integration in a mouse hematopoietic precursor cell line, show that transcribed retroviral integrations can lead to microscopically detectable changes in large-scale chromatin structure. This includes a repositioning to a more internal nuclear position and a decondensation of neighboring chromosomal sequences.
